# Muon Irradiation of ZnO Rods: Superparamagnetic Nature Induced by Defects

**DOI:** 10.3390/nano12020184

**Published:** 2022-01-06

**Authors:** Cody Landry, Alexander Morrison, Mehdi Esmaeili, Khashayar Ghandi

**Affiliations:** Department of Chemistry, University of Guelph, 50 Stone Road East, Guelph, ON N1G 2W1, Canada; clandr03@uoguelph.ca (C.L.); amorri27@uoguelph.ca (A.M.); esmaeili@uoguelph.ca (M.E.)

**Keywords:** nanostructures, magnetism, physical chemistry, nanochemistry, superparamagnetism

## Abstract

In this work, through a combination of photoluminescence spectroscopy, X-ray powder diffraction and magnetic measurements, it is determined that ZnO rods, made hydrothermally using a combination of magnetic field with respect to the force of gravity, exhibit superparamagnetic properties which emerge from Zn defects. These Zn defects result in a size-dependent superparamagnetic property of the rods. Red emissions, characteristic of Zn vacancies, and magnetic susceptibility both increased with decreasing rod size. The ZnO rods have significantly larger superparamagnetic cluster sizes (one order of magnitude) and lower fluctuation rates when compared to other superparamagnetic particles.

## 1. Introduction

Magnetic nanomaterials find application in a wide range of medical and industrial contexts, seeing potential use as, for example, a heterogeneous catalyst [1,2], an inductive median to convert electromagnetic energy into heat [3], a component in data storage [4], as sensors to detect infectious diseases [5], in ferrofluids [6], MRI agents [7], and in drug delivery [8]. The types of magnetic material used are as diverse as their applications, even non-magnetic nanomaterials can be made magnetic by doping [9] or by creating defects in their structures [10]. Given the wide range of such functional magnetic materials and their applications, there is a great incentive to investigate the magnetic character of many materials to inform the efficient development of technologies [11].

Recently, we have shown that ZnO rods exhibit size-specific magnetic properties. Using an interplay of an externally applied magnetic field and gravity, we were able to control the morphology of the ZnO rods, allowing for the structure, and its magnetic properties, to be tuned to a specific application [5]. In this work we have shown that our ZnO rods exhibit superparamagnetic properties. Superparamagnetic material show strong potential in medical applications, particularly in treatment of cancer and medical imaging [12]. Superparamagnetic iron oxide nanoparticles (SPIONs) have attracted attention in the medical field for several decades in targeted drug delivery, radiotherapy, and imaging [12]. Other materials, such as ZnO nanomaterial, have the potential to be alternatives if they could be made superparamagnetic [5,13,14,15].

To conceptualize how morphological properties of the material affect their magnetic properties, we look to a brief description of how the distribution and character of domains within a superparamagnetic material influence the magnetic character of the material. Below the Curie temperature, we may consider an individual superparamagnetic nanoparticle as being composed of i magnetic subdomains consisting of n dipoles of magnetic moment $m_{s}$ exhibiting complete spin polarization, i.e., each subdomain acting as an independent dipole carrying a magnetic moment $m_{d}$ with a magnitude directly proportional to the volume V of the subdomain,

(1) $$m_{d}=n_{i}m_{s}\propto Vm_{s}.$$

Each nanoparticle is unmagnetized and thus carries a bulk magnetization M of zero, i.e.,

(2) $$\underset{i}{\sum }m_{i}=M\;\approx 0.$$

The energetics of an individual subdomain is broadly influenced by two factors [12]: structural anisotropies and magnetic coupling

(3) $$E=\left[E_{\mathrm{Structural}}\right]+\left[E_{\mathrm{Coupling}}\right],$$

yielding a potential barrier between magnetic states

(4) $$\Delta E=E_{↑}–E_{↓}$$

Each deviation from a perfectly isotropic system results in an axis which is more energetically favorable for the spin polarization to lie along. The largest contributions include the shape of the subdomain, internal tensile forces, i.e., stress and strain, and deviation of the polarization from the most energetically favorable axis as determined by the crystal structure, namely, the energy of the magnetic anisotropy:

(5) $$E_{\mathrm{Structural}}=E_{\mathrm{Shape}\;}+E_{\mathrm{Tensile}}+E_{\mathrm{Magnetic}\;\mathrm{Anisotropy}}.$$

Each domain also couples to an external field $B_{\mathrm{ext}}$ as well as the other  i subdomains,

(6) $$E_{\mathrm{Coupling}}=\left[E_{\mathrm{External}}\right]+\left[E_{\mathrm{Interdomain}}\right],$$

the net effect being, for a given external field, there is a potential barrier between spin states of a given subdomain. A spin-flip of a subdomain will not be spontaneous unless there exists sufficient thermal energy to overcome this barrier;

(7) $$kT\geq \;E_{↑}–E_{↓}$$

that is, there exists a temperature above which a spin-flip will become spontaneous.

In single ferromagnetic or ferrimagnetic domains, the magnetic moments come to an order below the Curie magnetic transition temperature. The spontaneous magnetization directs the array of magnetic moments in each cluster along the “easy axis”. An easy axis is a crystallographic axis defined by the coupling of electron spin and orbital angular momentum at a lattice point. These couplings are the source of the anisotropy energy.

**Figure 1:**
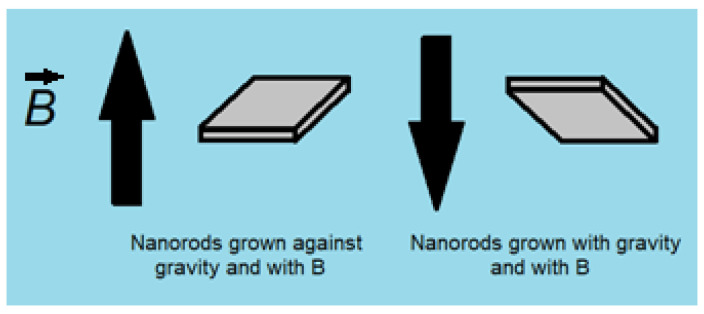
Experimental setup used for ZnO rod growth. All samples discussed in this article were synthesized against gravity unless otherwise specified.

ZnO is typically thought to be diamagnetic (non-magnetic) based on its electron configuration; however, ZnO nanostructures have been shown to exhibit magnetic behavior, the origin of which is still an active matter of debate [5,15,16,17,18,19,20,21]. To resolve this curiosity, computational investigations into the magnetic properties of ZnO nanostructures have been carried out in order to form a hypothesis for the origin of this mysterious magnetism [16,17,18,19,20,21,22]. Magnetism caused by defects, such as Zn vacancies and grain boundary defects, have all been shown to have computational validity. However, to date, there has been little experimental effort to substantiate such ideas. This work aims to begin to fill this gap. We seek to answer the following questions: what types of defects exist in magnetic ZnO rods grown hydrothermally under differing external field conditions shown in Figure 1? How would the defects in such rods affect their magnetic properties, if any? To what extent do the magnetic properties of the rods impact their morphology and vice versa? How can we take advantage of magnetic field during the synthetic process to change the properties of ZnO rods? To answer these, we will attempt to correlate experimental data with the available theoretical results [5].

## 2. Materials and Methods

### 2.1. Synthesis

The ZnO rods were synthesized on an indium tin oxide (ITO) (Sigma-Aldrich Canada Co., Oakville, ON, CA) substrate at 90 °C for 2 h in an aqueous solution of zinc nitrate hydrate (0.025 M) (Sigma-Aldrich Canada Co., Oakville, ON, CA) and hexamethylenetetramine (0.025 M) (Sigma-Aldrich Canada Co., Oakville, ON, CA). Sample 1 was grown in an 850-gauss magnetic field and sample 2 was grown with no magnetic field to produce different sizes of rods. All syntheses were conducted according to Figure 1 which shows a schematic of the experimental setup that allows for the use of magnetic fields and gravity to influence the growth of the ZnO rods. The direction of the substrate (up or down) determines whether the ZnO rods grow with the direction of gravity or against. A donut shaped 0.1 T permanent magnet was used to supply a magnetic field and the distance from the magnet determined the strength of the field at the substrate during ZnO rod growth.

### 2.2. Conventional Characterization Methods

Electron micrographs were taken using a JEOL 2000 scanning electron microscope (SEM) (JEOL Ltd., Akishima, Tokyo) and photoluminescence (PL) using the Molecular Devices SpectraMax M5 (Molecular Devices, LLC., San Jose, CA, USA). The magnetic susceptibility measurements were performed using the Physical Properties Measurements System (PPMS) (ORNL, Oak Ridge, TN., USA). The X-ray photoelectron spectroscopy (XPS) measurements were performed using the VG microtech Multilab ESCA 2000 System (Thermo Fisher Scientific, Waltham, MA, USA). The X-ray powder diffraction was taken using an Empyrean PANalytical X-ray powder diffractometer (Malvern Panaltyical Ltd., Malvern, UK.)

### 2.3. μSR

Muon spin relaxation (μSR) uses positive muons (henceforth: muon), a spin ½ lepton with 1/9th the mass of a proton, to measure the electronic and magnetic properties of matter. The positive muon has a large gyromagnetic ratio of γ_μ_ = 2π × 13.553882 kHz G^−1^, making it sensitive to internal magnetic fields [23,24,25,26,27].

The muons are created through the decay of pions and are 100% spin polarized when implanted into matter. Nearly all the muon decays are detected making it a sensitive technique for studying local environments. When muons are implanted in the sample, their spin precesses around the local magnetic fields. When the muon decays after 2.2 μs, it emits a positron, preferentially in the direction of its spin. The asymmetry of the positron emission (from forward and backwards detectors) creates a muon signal that can be interpreted by fitting to theoretical models [23,24,25,26,27].

In total, 0.3 g of each sample was collected and placed into custom sample holders made of Mylar to perform μSR experiments. The μSR experiments were performed at TRIUMF in Vancouver, Canada over three beam schedules. The Beamline we used was the M20D line, LAMPF (TRIUMF, Vancouver, BC, CA). We used the VG-Quant: Gas Flow Cryostat—Quantum Technology with the μ-Veto adaptor (TRIUMF Vancouver, BC, CA). Surface muons with momentum of 29 MeVc^−1^ were used in these measurements. In this study, we probed the local magnetic properties of ZnO rods using a method, called zero-field μSR (ZF), in which the magnetic field of the earth is corrected for, thus putting the sample in a true zero-field environment.

## 3. Results and Discussion

### 3.1. Morphology

**Figure 2:**
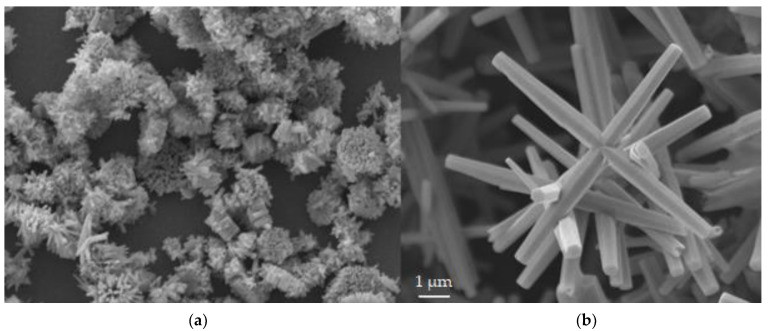
(**a**) SEM image for sample 1; (**b**) SEM image for and sample 2, for ZnO rods grown on ITO. Sample 1 shows bundles of small rods. Sample 2 yields a combination of larger rods and “nanoflowers”. Both Images are at the same scale.

The SEM results are shown in Figure 2 for sample 1 (a) and sample 2 (b). The average dimensions of the sample 1 ZnO rods are [5]: 0.66 ± 0.11/0.17 ± 0.05 μm for length/width, respectively. The average dimensions of the sample 2 ZnO rods are [5]: 9.2 ± 1.9/1.8 ± 0.5 μm for length/width, respectively. Abbreviations for synthetic conditions are as follows: NF (no magnetic field), ag (against gravity), and wg (with gravity). Samples 1 and 2 were synthesized “ag” at 850 and 0 G, respectively.

### 3.2. Photoluminescence/X-Ray Photoelectron Spectroscopy

**Figure 3:**
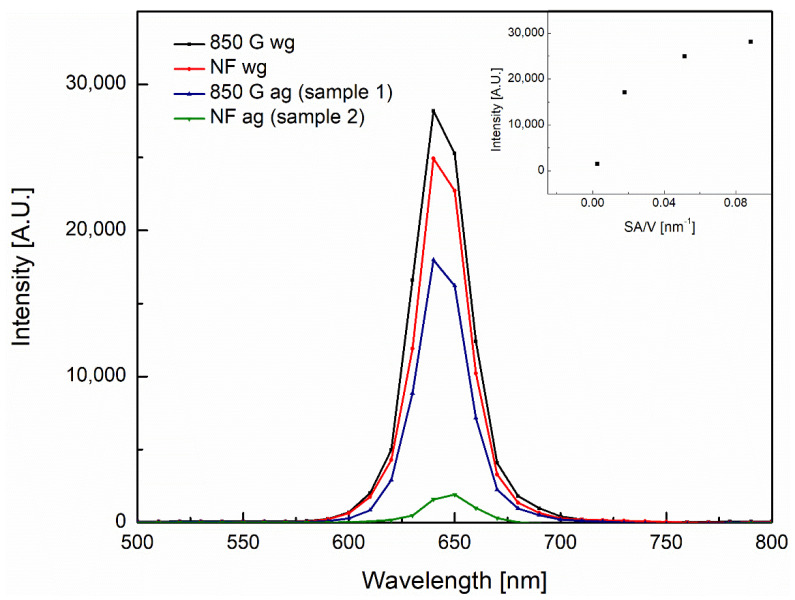
PL of different morphologies of ZnO rods. 850 G applied field wg, the smallest rods, have the greatest intensity; NF ag, the largest rods, have the smallest intensity. The inset shows the maximum intensity versus the surface area to volume ratio on the x axis.

PL as well as XPS (Figure 3, Figures S1 and S2 in supplementary information) were used to characterize defects in the ZnO structure. Figure 3 shows the PL spectra for sample 1 (NF ag) and sample 2 (850 G ag). Other samples (850 wg and NFwg) [5] are shown in order to study the effect on the PL red emissions as a function of particle size.

The PL spectra show a red emission which indicates the presence of zinc vacancies [21]. Other defects emit strongly in lower wavelengths and grain boundary defects emit broad bands [22]. The emission profile demonstrates that only zinc vacancy defects are present in any noticeable quantity [22]. The inset of Figure 3 shows that as the size of ZnO particles decrease, the photoluminescence intensity increases. There is a clear trend between the red photoluminescence peak intensity and the surface area to volume ratio (SA/V) that trends towards a maximum with increasing SA/V.

The XPS data are shown in Figures S1 and S2, for samples 1 and 2, respectively, in the supplementary information. There was considerable charging of the sample which caused a shift in the binding energies. This was corrected using the C1s adventitious peak at (284.8 eV) by subtracting 9.8 eV from the recorded binding energies for sample 1 and 10.2 eV for sample 2. The peaks at 1042 and 1019 eV represent Zn 2p_1/2_ and Zn 2p_3/2_ peaks, respectively, for both Figures S1 and S2. The splitting of the peaks is 23 eV for sample 1 and 23.3 eV for sample 2 which is typical for ZnO nanostructures [28]. For the oxygen XPS, the peak at 529 represents O 1s. The oxygen signal is a combination of Zn-O and Zn-O-H (from the surface) species which appears as a shoulder on the main Zn-O peak [28]. The relative abundances show that the concentration of oxygen (Figure S1a) for sample 1 is 50% larger than that of the zinc (Figure S1b). Comparatively, sample 2 shows a 35% greater concentration of oxygen (Figure S2a) than Zn (Figure S2b). The peak in the oxygen spectrum near 520 eV are due satellite peaks caused by x-ray source being non-monochromatic. Considering that XPS can only probe 10 nm deep into a sample, it is clear that Zn vacancies do exist at least near the surface of the rods. Additionally, there is a larger number of vacancies in sample 1 compared to sample 2. This is consistent with the magnetic data described later.

The spectra for samples 1 and 2 are almost identical for the O 1s and Z 2p spectra in both shape, and binding energy values within a reasonable variance, <1 eV. The binding energies for the Zn 2p peaks in our samples are approximately 2 eV lower than typical ZnO nanostructures [28]. This shift to lower binding energy maybe be due to slight differences in the chemical environment on the surface of our rods that cause this shift since our samples were not annealed after being synthesized. The annealing process is typical after synthesis of nanoparticles to remove defects; however, it was desirable to retain the defects in our structure to study the magnetic properties. Although there are differences in the relative abundances of oxygen and Zn for our samples, qualitatively consistent with the difference in the magnetic properties, the fact they their XPS spectra shapes are very similar indicates that most of the cause for the differences in their magnetic properties lie in the bulk not the surface properties. This can also be seen when we compare the XPS data with the PL data, which shows a much larger difference in the vacancies in the two samples. This is further expanded in our discussion of the magnetic properties.

### 3.3. X-ray Diffraction

Small pieces of ITO films covered by ZnO rods were placed on a spin stage on an Empyrean PANalytical X-ray powder diffractometer. The employed diffractometer included a radiation source of Cu Kα1 (λ = 1.54184 Å) with NiKβ filter and PIXel1D linear detector, designed in reflection geometry. The diffraction patterns were recorded in the 5–80° 2θ range. Data were collected and analyzed with the Data Collector [29] and HighScore Plus [30] software.

**Figure 4:**
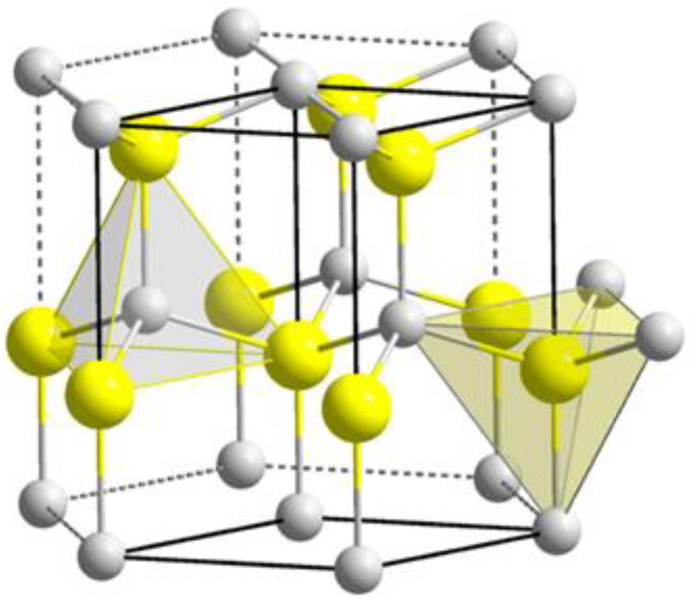
ZnO ionic alignments in a wurtzite crystal structure, Adapted from Ref. [30]. Yellow represents zinc and grey oxygen.

**Figure 5:**
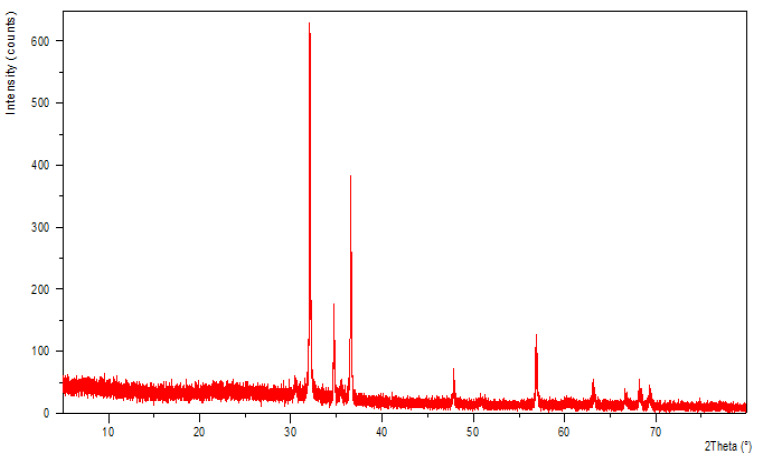
ZnO PXRD pattern at room temperature.

ZnO crystallizes in a wurtzite hexagonal close packing (hcp) arrangement (Figure 4). The ZnO crystals have the *P6_3_mc* space group, and the unit cell parameters of a and b equal 3.2495 Å and c equals 5.2069 Å (Figure 4 and Figure 5). In the ZnO crystal structure, all Zn^2+^ cations form the cp layers while the O^2−^ anions occupy all the interstitial tetrahedral (T^+^) sites [29]. Therefore, all the interstitial tetrahedral (T^−^) and octahedral (O) sites remain unoccupied. As the two highlighted tetrahedral geometry shapes indicate, each Zn^2+^ cation is coordinated by four O^2-^ anions in the same way as each O^2−^ anion coordinated by four Zn^2+^ cations, forming tetrahedrons (Figure 4) [30].

Analyzing the ZnO crystal structure and finding the correlation between ions in the interstitial sites can help in explaining the properties of this compound. For instance, the vacant sites can be randomly filled with zinc or oxygen elements or impurities in a point defect structure [31]. Defects, by their nature, can partially transform a crystalline sample into an amorphous phase. An XRD pattern with a partial amorphous phase is different from the perfect crystal’s pattern. By comparing the XRD patterns of our synthesized ZnO with the reported patterns in the literature, the nature of defects in our crystals was investigated. Our analysis further confirms that defects are available in our ZnO samples, as the obtained PXRD pattern (Figure 5) is like the reported patterns in the papers for crystal structures with point zinc defects [32,33]. Using on the Scherrer equation, we found that the crystallite grain size is 57.66 nm based on the (101) peak at 36.5504 (°2θ) which had a FWHM of 0.1502 (°2θ). Calculating the grain sized based on the (100) peak gave a similar grain size.

### 3.4. Magnetic Susceptibility

**Figure 6:**
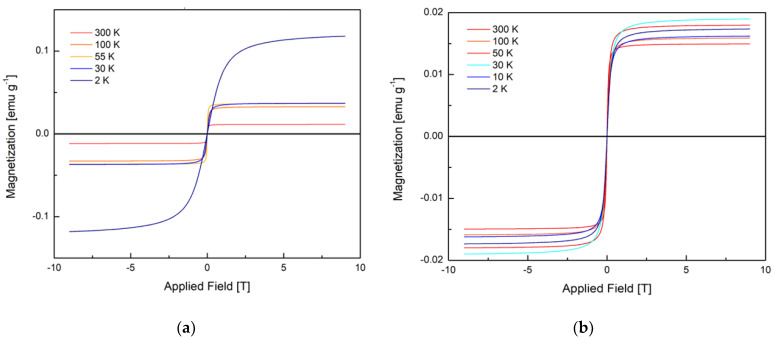
(**a**) Magnetization curve of sample 1 (smaller rods) from 2 to 300 K; (**b**) magnetization curve of sample 2 (larger rods) from 2 to 300 K. It is important to note the difference in the magnitude of the magnetization between samples.

The bulk susceptibility and magnetization were measured from 2 to 300 K for both samples. All measurements were adjusted using a diamagnetic correction factor to consider the diamagnetic signal from both the sample holder and the material. The magnetization curves for samples 1 and 2 shown in (Figure 6a,b respectively) display a non-linear dependence of magnetization as a function of applied field between 2 and 300 K.

Based on the sigmoidal shape of the curves and the lack of hysteresis (except at 2 K) the magnetic properties of the samples fit best a superparamagnetic model and therefore these curves are fit to the classical Langevin model shown in Equation (8) [23],

(8) $$M\left(B,\;T,\;\mu \right)=M_{s}\left\{coth\left(\frac{\mu B}{k_{B}T}\right)-\frac{k_{B}T}{\mu B}\right\}$$

the Langevin model is a test for the quality of the superparamagnetic domains where *M* is the magnetization (emu/g) *M_s_* is the saturation magnetization (emu/g), μ is the magnetic moment (J/T), *T* is temperature (K), *B* is applied field (*T*), and *k_B_* is the Boltzmann constant (m^2^ kg s^−2^ K^−1^). The fit parameters for each curve are listed in Table 1.

The quality of a superparamagnetic material is ‘good’ when there is no spatial overlap between magnetic domains [23]. The model fits well for both samples across all temperatures except for 2 K. For sample 1, the maximum magnetization increases as the temperature decreases which is typical, and at 2 K the maximum magnetization increases significantly, which may indicate ferromagnetic-like interactions [24]. For sample 2, however, the maximum magnetization goes through a maximum at 30 K. It is possible that the superparamagnetic domains in this case are exhibiting antiferromagnetic-like interactions below 30 K [25]. Another possibility is that these interactions are indicative of phase transitions; however, the temperature-dependent magnetization does not support this, as described below. Additionally, comparing the magnetization curves of both samples, we see that the degree of magnetization of sample 1 has stronger response to an applied field by almost one order of magnitude. This is a significant result as it further indicates that the magnetic properties of our sample could be tuned significantly with small changes to the morphology brought on by changes of external magnetic field during synthesis.

From the Langevin fits, the superparamagnetic domain moment at 300 K may be extracted. We find this value in samples 1 and 2 to be 22,573 and 12,989 μ_B_, respectively. Such values are large compared to other measured superparamagnetic domain moments [22,23,24]. This suggests that the magnetic properties arising from defects observed here may be mechanistically distinct from those caused by doping with magnetic atoms [25,26]. The moment is even greater in magnitude than small, purely superparamagnetic particles [25].

**Figure 7:**
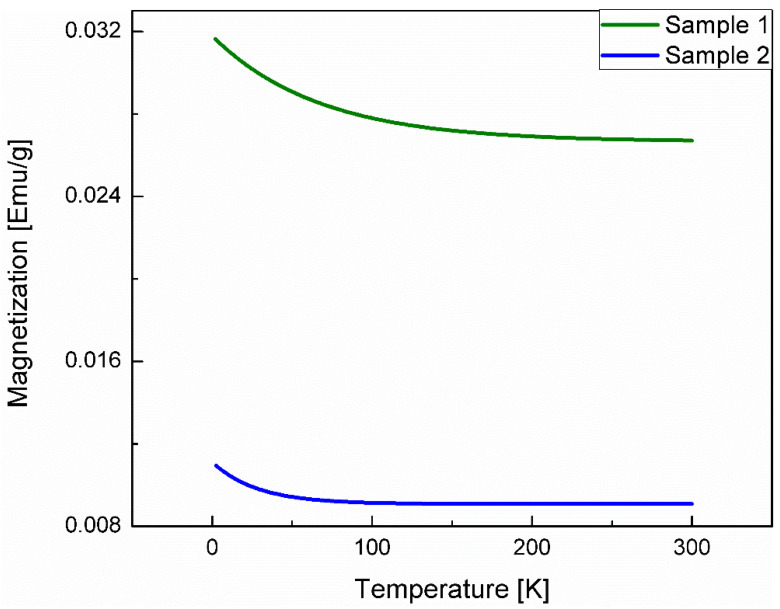
Temperature-dependent magnetization at 0.1 T from 2 to 300 K for sample 1 (green) and sample 2 (blue). Neither sample shows any sharp phase transition above 2 K. Similar to the B–H curves, sample 1 has greater magnetization than sample 2.

Figure 7 shows the temperature-dependent magnetization of sample 1 (green) and sample 2 (blue). The temperature-dependent magnetization shows no obvious phase transition within the temperature range probed and displays a paramagnetic-type dependence, typical of superparamagnetic materials [10].

A field strength of 0.1 T was chosen for the temperature-dependent magnetization measurement as this field shows the most consistent magnetization across both samples. Considering magnetic phase transitions show more prominently at lower applied fields, a phase transition is unlikely to be the cause for the temperature dependence of the maximum magnetization in the B–H curve [26].

From the diamagnetic slope corrections, the diamagnetic susceptibility may be computed. We find this value to be −3.19 × 10^−7^ emu G^−1^ g^−1^ for sample 1 and −5.69 × 10^−7^ emu G^−1^ g^−1^ for sample 2. It is understandable that the diamagnetic susceptibility for both samples are close in magnitude as the diamagnetic component in both samples is the same—ZnO with no defects.

### 3.5. Zero-Field μSR

**Figure 8:**
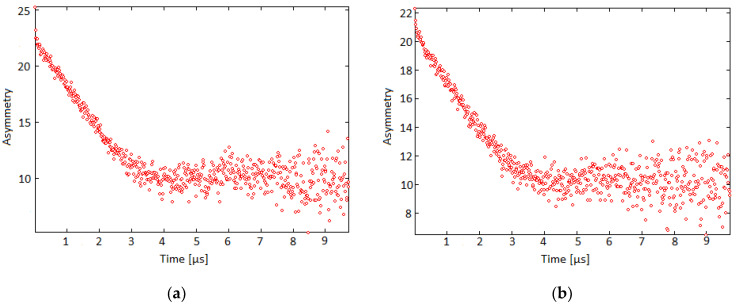
Time-dependent asymmetry for (**a**) sample 1 at 2.1 K; (**b**) sample 2 at 2.4 K. Both curves were fit with a non-relaxing component, a relaxing Lorentzian component, and a stretched exponential Kubo–Toyabe.

In this study we take advantage of the large gyromagnetic ratio of the muon (*γ_μ_* = 2π × 13.553882 kHz G^−1^), to probe the magnetic properties of our ZnO rods with ZF-μSR. The raw data at 2 K for samples 1 and 2 are shown in Figure 8.

ZF is a method to precisely probe the local magnetic structure of a sample. In this case, when a muon is implanted in the sample, the spin of the muon precesses around the local magnetic field within the material. In a highly organized, ferromagnetic (or antiferromagnetic) sample this would result in a single oscillating component as can be seen in single crystal Sr_2_CuO_2_C1_2_ [34,35]. In samples with more disordered magnetic components, however, there are many different muon precession frequencies which results in a relaxation curve [34]. The shape of the curve depends on a variety of factors including dynamics, strength of the magnetic moment at the stopping site, and the distribution of the magnetic moments throughout the material [35]. The ZF data for samples 1 and 2 were analyzed using a three-component fit (Equation (9)) consisting of: a zero-field Kubo–Toyabe function multiplied by a root exponential, a Lorentzian relaxing component, and a non-relaxing component,

(9) $$P\left(t\right)=A_{RE}\left(\frac{1}{3}+\frac{2}{3}\left(1-{\gamma_{\mu }}^{2}\Delta^{2}t^{2}\right)e^{\left(\frac{-1}{2}{\gamma_{\mu }}^{2}\Delta^{2}t^{2}\right)}\right)e^{{\left(-\lambda t\right)}^{\beta }}+A_{L}e^{-\lambda t}+A_{NR}$$

where *A_RE_* is the amplitude of the root exponential component, Δ is the variance in the average magnetic moment felt by the muon, or the Gaussian field width (G), *λ* is the relaxation rate, *t* is time (μs), *β* is the dimensionless root exponential which takes into account motional narrowing caused by fluctuating superparamagnetic moments [35], and *A_NR_* is the amplitude of the non-relaxing (diamagnetic) component. The stretch factor was 0.505 (±0.004) for sample 1 and 0.501 (±0.005) for sample 2. The three-component fit is consistent with three different average muon stopping environments: *A_RE_*: the muons stop very close to the magnetic defects and relax due to fluctuating superparamagnetic moments; *A_L_*: muons stop near the magnetic defect and react either via electron exchange or electron transfer with the unpaired electrons delocalized in the defect. It should be noted that we cannot rule out the electron transfer from electrons generated by muon irradiation (radiation induced interactions) to muons, as a source of this reaction. *A_NR_*: the muons stop in a diamagnetic environment and do not relax. Such a multi-component fit is typical of dilute magnetic structures [27].

The Kubo–Toyabe oscillation indicates that there is order in our sample consistent with the ferromagnetic domains; however, since those ferromagnetic domains experience spin fluctuations, the characteristic Kubo–Toyabe “dip” is much shallower than what would be expected in a static system [35].

Other possible explanations for the dynamic nature of the system may be muon hopping, a phenomenon in which the muon, once implanted, will diffuse across the lattice experiencing multiple sites in one lifetime. However, if this were the case, we would have expected to observe a change from root exponential to a simple exponential with increasing temperature [36]. This is not present in our measurements, suggesting that a given muon is trapped in a low energy position; most likely a bonding centered position as has been found with muonium (muon plus e^−^) in bulk ZnO [37]. Furthermore, based on the root exponential form remaining constant, it can be interpreted that the superparamagnetic moments are of similar size and are fairly monodispersed, otherwise we should have observed a significant decrease in the stretch factor from *β* = 0.5 to a smaller value [38].

The root exponential form is caused by the averaging of different fluctuating local fields at different muons sites as one would expect in a random dilute superparamagnetic system [27,39]. Based on the fit to the relaxation curve similar to that form used by Bewley and Cywinksi to describe superparamagnetic species [27], it is reasonable to say that the environment experienced by the muon is similar to that of a superparamagnetic system, thus the dynamics can be studied in a similar fashion.

**Figure 9:**
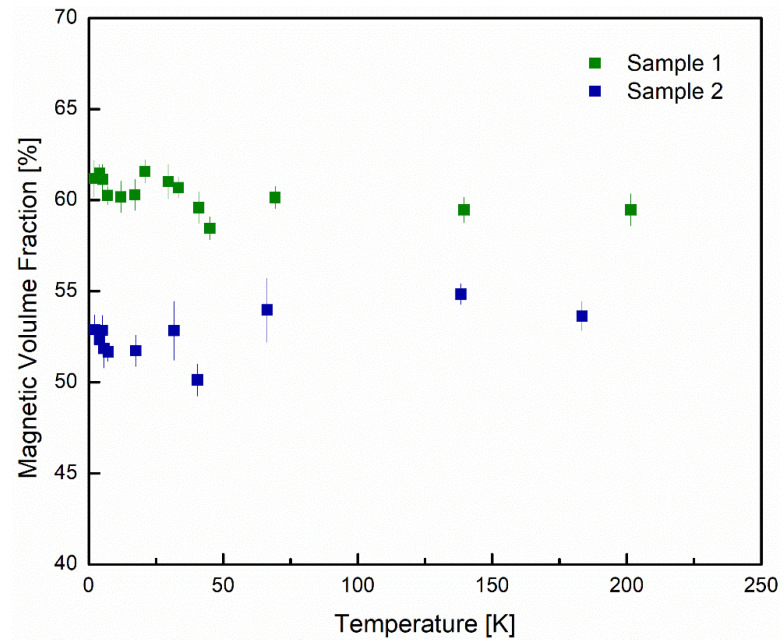
Percent magnetic volume fraction for samples 1 (green) and 2 (blue) with increasing temperature. The average percent magnetic fractions for samples 1 and 2 are 65% and 74%, respectively.

Figure 8a shows the time-dependent asymmetry for sample 1 at 2.1 K. For sample 1, the average percent volume fraction of the magnetic component is 60.3 (±0.8)%, the average field felt by the muons is 4.77 (±0.15) G and the Gaussian field width is 0.3486 (±0.0005) G. Figure 8b shows the time-dependent asymmetry for sample 2 at 2.4 K. For sample 2, the average percent volume fraction of the magnetic component is 52.6 (±1.3)%, the average field felt by the muon is 2.47 (±0.55) G and the Gaussian field width is 0.3254 (±0.0004) G. The fast relaxation observed in sample 1 is much larger than that of sample 2 which means that more muons in sample 1 react with electrons which is consistent with the evidence that there are more defects in sample 1. Figure 9 shows the volume fraction as a function of temperature of sample 1 (green) and sample 2 (blue) over a wide temperature range.

**Figure 10:**
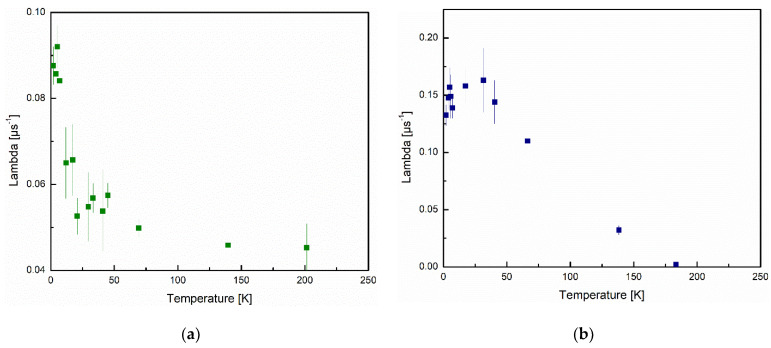
Relaxation rate of the stretch exponential component of the ZF fit for (**a**) samples 1; (**b**) sample 2, as a function of temperature.

The observation that there is no dramatic change in the volume fraction of either sample, confirms there is no phase transition within the measured temperature range [23,24]. The larger volume fraction of the magnetic component of sample 1 is consistent with the bulk magnetization measurements that show larger magnetic moments as well as larger saturation magnetizations in sample 1. Figure 10 shows the relaxation rates for the root exponential for samples 1 and 2 (Figure 10a,b respectively).

Sample 2 has a larger relaxation rate at all temperatures indicating that the spin fluctuation rate is higher for sample 2. This is potentially because the superparamagnetic domains are smaller, consistent with a smaller magnetic volume fraction. The relaxation rate for sample 1 decreases as a function of temperature monotonically; however, in sample 2 we observe an increase from 2 to 30 K followed by a decrease as temperature rises. Both of these trends are consistent with the trend of the maximum magnetization as a function of temperature for the B–H (magnetization as a function of applied field) curves of each sample. The fluctuations can be analyzed using the Néel–Arrhenius equations for superparamagnetic material (Equation (10))

(10) $$\nu_{N}=\nu_{0}e^{-\frac{E_{a}}{k_{B}T}}$$

where *ν_N_* is the average domain spin flip frequency (s^−1^), *ν*_0_ a prefactor called the intrinsic fluctuation frequency (s^−1^) [26], *E_a_* is the activation energy for the fluctuation (K), *T* is temperature (K), and *k_B_* is the Boltzmann constant (J K^−1^). It should be noted that, as in the case of Bewely and Cywinski [26], the independent variable is *T*ln*λ* and thus *ν_N_* must be broken down into its components. The domain flip frequency is the average fluctuation rate of the magnetic field felt by the muon and is related to the gyromagnetic ratio (*γ*), the relaxation rate (*λ*) and Gaussian field width (∆) of (Equation (9)) via Equations (11) and (12) [26],

(11) $$\nu_{N}=\frac{4a^{2}}{\lambda }$$

(12) a=Δγ

Based on the fit from the Kubo–Toyabe function, we find that *α* is 0.2968 (±0.0004) μs^−1^ and 0.3019 (±0.0008) μs^−1^, for samples 1 and 2, respectively. Our *α* values for both samples are lower than that of Bewely and Cywinski’s sample, which consisted of Cu disks doped with 2% Co. The defects that cause the magnetic moment in our ZnO rods are more homogenously distributed compared to the magnetic moments in their system, leading to a smaller variance between individual stopping sites.

Based on these α values, the intrinsic fluctuation rates for samples 1 and 2 are, respectively, 7.4 (±0.38) MHz and 6.4 (±0.63) MHz. These values for the intrinsic fluctuation rates are much lower than that found by Bewely and Cywinski, who found an intrinsic fluctuation rate of 0.7 and 2.7 GHz for their samples [27]. This much smaller fluctuation rate is the reason for much larger cluster relaxation time in our system compared to 0.1 to 1 ns reported based on classical superparamagnetic materials [40]. Possible reasons for this are discussed in the following paragraphs.

The activation energy for the superparamagnetic cluster fluctuation was 5 (±1.9) and 21 (±2.7) K, respectively, for samples 1 and 2. Comparison of other work including that of Bewely and Cywinski can be found in Table 2 [23,27,40]. The low activation energy, in particular for sample 1, suggests that the superparamagnetic moments are spin flipped easier compared to those superparamagnetic moments composed of single magnetic moments localized on atoms. Based on computational work on magnetism caused by Zn defects, it is shown the spin polarization is small, and is delocalized over 4 oxygen atoms [21]. It should also be noted that the electrons in the Zn defect are p orbital electrons, as opposed to d orbital electrons in magnetic metals, making the orbital angular momentum smaller.

Furthermore, in the case of delocalized electrons, the coupling between spin angular momentum and orbital angular momentum is smaller. This lower coupling of course lowers the total angular momentum and thus lowers the total magnetic dipole moment of the defect, making the moment easier to flip.

Regarding the low activation energy, it is useful to compare the volume of the cluster *V* and the anisotropy constant *K* where *KV* = *E_a_*. The volume of the superparamagnetic cluster can be calculated using Equation (13),

(13) $$V=V_{d}\frac{\mu }{\mu_{d}},$$

where *V_d_*, is the volume of the defect, *μ* is the magnetic moment of the superparamagnetic cluster found from the Langevin fit and *μ_d_* is the magnetic moment of the defect. Based on the nature of the defect as described by Yun et al. [21], the average Zn-O bond length found for similarly sized ZnO nanostructures (1.967 A) [41], and using a spherical approximation for the shape of a single magnetic defect, we find the volume of a defect to be 3.19 (±0.02) × 10^−29^ m^3^. The superparamagnetic cluster volumes for samples 1 and 2 are 4.05 (±0.02) and 2.33 (±0.02) × 10^−25^ m^3^, respectively, which is ~10-fold larger than that found in iron nanoclusters [23]. Using the average volume of our cluster and the activation energy we find that the anisotropy constant *K* is 153 (±64) and 1235 (±161) J m^−3^_._ These values are lower than those found in other superparamagnets [23,40]. The lower anisotropic constant is also consistent with the superparamagnetic moments being distributed approximately uniformly, which was based on the fitting parameter, *β* = 0.5 in the μSR data fit, as well as the lower ν_0_ value. It is also likely that the small deviations from the Langevin model used to fit the magnetic susceptibility data are due to small cluster interactions rather than anisotropy of cluster shape and distribution as the low *K* implies.

The size of the superparamagnetic cluster for the iron nanoclusters is used as a measurement for the iron nanocluster size itself, meaning that 100% of the cluster is magnetic [22]. However, in the case of the ZnO rods, only ~60% and ~50% of the rods in samples 1 and 2, respectively, are magnetic. Considering that our rods are significantly larger than the calculated superparamagnetic cluster volume, it means that our ZnO rods contain multiple superparamagnetic cluster domains and that they are spread throughout the rod homogeneously, based on both the bulk susceptibility and the μSR data. There is also a disproportionate amount of domains in sample 2 when compared to sample 1, as the cluster size in sample 2 is approximately half that of sample 1 but the % magnetic fraction is only 20% smaller in sample 2.

Considering sample 1 has a significantly higher average surface area to volume ratio than sample 2, the disproportionate magnetic volume fraction in sample 2 indicates that the defects are preferentially located in the bulk (not close to the surface). We also find that the anisotropy constant *K* value is approximately one order of magnitude smaller for sample 1, meaning that the clusters in sample 1 have more of a cubic symmetry which leads to the smaller activation energy. Based on Equations (3)–(6), this activation energy depends on the sum of the structural and coupling energies. There is no reason to believe that internal coupling between magnetic defects would be different between samples 1 and 2 therefore it is likely that structural effects result in the differences between the samples. Likewise, within the structural component there is no reason to believe that *E*_tensile_ would be different between samples 1 and 2 as it depends on the properties of the crystal structure which should be the same for both samples, as ZnO rods, regardless of size, have a typical wurtzite structure [42]. *E*_Shape_ and *E*_Magnetic anisotropy_ both depend on the properties of the superparamagnetic clusters. The lower anisotropy constant and thus lower *E*_Shape_ for sample 1 is most likely a result of the difference in the synthetic method. Having grown the rods in an 850 G magnetic field for sample 1, there is an added external field coupling component for the *E*_Coupling_ for Equation (3) which may cause the magnetic defects to cluster together rather than spread out randomly for sample 2 relative to sample 1, causing the shape of the defect to take on a more symmetric structure in sample 1. It is also likely the case that the *E*_Magnetic anisotropy_ is lower for sample 1 since the magnetic field applied during the synthesis was along the direction of growth of the ZnO rods. This created a magnetic field vector along which defects in the cluster could orient their moments during the assembly, causing the superparamagnetic moment of the clusters to be oriented preferentially along that direction of growth which is also the most energetically favorable axis.

Considering that our intrinsic fluctuation rates are significantly lower than those found in other superparamagnetic material, and indeed they fall out what would typically be expected in a classical superparamagnetic, our vacancy defect magnetism seems to be unique. If we consider Equations (10) and (11), the *E_a_* should have a significant effect on the *v*_0_, being in the exponent, with less contributions from *λ* and *α*. Certainly the *E_a_* is smaller for samples 1 and 2, by a factor of 7 and 0.7-fold larger, respectively, from the smallest values in our comparison, this does not account for the three orders of magnitude difference in *v*_0_, at least in sample 2 [27]. Of course, we also found that our alpha value was small, but only by approximately one order of magnitude from that found by Bewely and Cywinksi and not all that different from that found in Jackson et al. (*σ* = 0.4 μs^−1^) [23,27]. However, the *σ* found by Jackson et al. was found from a Lorentzian relaxation function rather than the Kubo–Toyabe function used by this work and that of Bewely and Cynwinksi, though they should still be able to be compared, as they simply represent variance in the magnetic field felt by the muon [23,27]. The most stark difference between our measurements and those found by Bewely and Cynwinski, and Jackson et al., is that within a comparable temperature range, they found that their relaxation rate changed by several orders of magnitude compared to ours seeing changes of, at most, a factor of 4. This of course significantly lowers the slope of the Arrhenius plot which affects the *v*_0_, that is, a small change in the relaxation rate *λ*, or fluctuation rate *v_N_*, as a function of temperature, results in a small *v*_0_.

Even though we are certain of the type of magnetism our ZnO rods display, and the source of the magnetism, we cannot rule out the possibility of confinement effects on how the two different sized ZnO rods differ in their specific magnetic parameters such as: cluster moment, intrinsic fluctuation, and anisotropy constant. It is found that much smaller ZnO nanoparticles (sub 100 nm in all dimensions) tend to exhibit ferromagnetic properties [20,43,44,45]. This could be a result of confinement effects as the nanoparticles exhibit stronger ferromagnetic properties as they become smaller [20,43,44,45]. It is believed that these ferromagnetic properties may originate from different types of surfactants [43], or grain boundary effects in thin layers [20]; however, this ferromagnetism is common among many different types of small ZnO nanoparticles and therefore it is plausible to assume it is an intrinsic property of the structure such as structural defects. A recent study investigating the size effects over long ranges, bulk to 36 nm particles [44], found that ZnO rods transition from diamagnetic, to paramagnetic, to ferromagnetic as the size decreases, further emphasizing that confinement effects may be at play. It was also found that synthesizing ZnO nanoparticles of the near-same size with different sources of Zn ions result in changes in the ferromagnetic properties of the resulting nanoparticles [45]. This may suggest that the magnetism originates from an intrinsic defect in the structure rather than simple surfactant modification or grain boundary effects. What is clear from our studies and comparison with other works discussed here, is the need for further future systematic investigations of the interesting magnetic properties of ZnO nanostructures and microstructures.

Considering the superparamagnetic nature of our nanomaterials and the degree in which we can control their size, there could be medical applications to which they can be applied. In terms of size, it is generally the case that larger sized ZnO nanoparticles are less toxic than smaller nanoparticles, with microsized rods being the least toxic [46]. This is thought to be due to the increased generation of reactive oxygen species (ROS) and Zn ions in the cell due to increased availability of surface area of smaller nanoparticles [46]. It is of course a more complicated situation, however, as the increased generation of ROS does not necessarily result in greater cell death [47]. It was found that both ZnO nanorods and nanospheres resulted in similar concentrations of ROS in cells; however, the nanospheres resulted in greater cell death [47]. To that end, larger microrods could have potential for drug delivery purposes while smaller nanorods could have potential to induce cell death in cancer cells [48]. The magnetic properties mean that these nanoparticles could be used in the localization process in the body and work independently (or simultaneously) as an MRI contrast agent, and be used to induce hyperthermia to kill cells [48].

## 4. Conclusions

This work provides evidence that zinc defects in the structure of ZnO rods are strongly related to the magnetic environment with a combination of bulk magnetic susceptibility, photoluminescence spectroscopy, X-ray photoelectron spectroscopy and μSR.

Through the use of XPS, XRD and PL, we show that our ZnO rods do in fact have defects and that they are Zn vacancies. Magnetic susceptibility measurements have shown that our ZnO rods exhibit superparamagnetic properties and that the smaller rods exhibit greater magnetization. This implies that there should be a higher concentration of magnetic defects in the smaller rods, which was also observed based on the PL intensity and XPS Zn and O abundances. These data agree with previous computational simulations that predict the magnetism is caused by Zn vacancies.

ZF-μSR was used to probe the local magnetic properties and shows that the local magnetic environment is of random dilute superparamagnetic system in nature. The volume fractions of the magnetic component for samples 1 and 2 were 60.3 (±0.8)% and 52.6 (±1.3)%, respectively, which is consistent with the larger bulk magnetization found for sample 1.

We have also shown that our superparamagnetic rods are unique compared to others found in the literature. The low activation energy and low intrinsic fluctuation rate provide explanations for why an applied field during ZnO rod growth has such a significant impact. The small intrinsic fluctuation rates are most likely due in part to the nature of the magnetic dipole moments which make up the cluster as well as the lower anisotropy constant and smaller dependence of fluctuation rates on temperature [39].

Our synthetic method offers considerable control over the size and magnetic properties of our ZnO rods. We have shown that, by applying a magnetic field during synthesis, we add an external coupling component to the coupling energy which promotes the formation of superparamagnetic clusters with cubic symmetry and relatively low magnetic anisotropy. This applied magnetic field acts on the defects as they form during synthesis, which become trapped due to the low temperature of the synthetic process. By changing the strength of the magnetic field, we can vary the external coupling component allowing us to control the outcome of the synthesis, both in terms of morphology and magnetic properties. This considerable control over the magnetic properties and morphology could be taken advantage of in the development of superparamagnetic material, especially for medical purposes [48].

## Figures and Tables

**Table 1:** Fit parameters for Langevin function.

	Sample 1			Sample 2		
*T* (K)	*μ* (J/T)	*M_s_* (Emu/g)	χ^2^	*μ* (J/T)	*M_s_* (Emu/g)	χ^2^
2	6.5 × 10^−23^	0.124	0.08982983	2.8 × 10^−22^	0.019	0.01756598
10	-	-	-	3.7 × 10^−21^	0.018	0.04717651
30	6.5 × 10^−21^	0.038	0.00548285	1.5 × 10^−21^	0.017	0.01950495
55	4.6 × 10^−20^	0.037	0.00375101	2.2 × 10^−20^	0.016	0.00132365
100	3.7 × 10^−20^	0.033	0.00052324	1.1 × 10^−20^	0.016	0.06141114
300	2.1 × 10^−19^	0.011	0.00363221	1.2 × 10^−19^	0.015	0.00048272

**Table 2:** Comparison of superparamagnetic cluster constants, for samples 1 and 2, respectively, from this work with other published data.

	Material	*v*_0_ (GHz)	*E_a_*/*k_B_* (K)	*K* (J M^−3^)	*V* (m^3^)
Present work	ZnO rods	7.36, 6.40 (10^−3^)	4.5, 20.8	153, 1235	4.05, 2.33 (10^−25^)
Bewley [26]	2% Co-doped Cu	0.7, 2.7	35.4, 48.4	-	-
Jackson [22]	Iron nanoclusters	833	51	2.3 × 10^5^	1.2 × 10^−26^
Frandsen [34]	Fe_3_O_4_ nanoparticle	36	101, 1750	19, 6.1 (10^3^)	-

## Data Availability

The data presented in this study are available on request from the corresponding author. The data are not publicly available due to cybersecurity. All the raw data is available on TRIUMF secure server.

## Supplementary Materials

The following supporting information can be downloaded at: https://www.mdpi.com/article/10.3390/nano12020184/s1, Figure S1: XPS of sample 1 ZnO rods. (a) shows the binding energy (eV) for the different oxy-gen species. (b) shows the binding energy (eV) for the different Zn species.; Figure S2: XPS of sample 2 ZnO rods. (a) shows the binding energy (eV) for the different oxy-gen species. (b) shows the binding energy (eV) for the different Zn species.
