# Supplementary material for: Muon Irradiation of ZnO Rods: Superparamagnetic Nature Induced by Defects
Source: Nanomaterials (Basel). 2022 Jan 6;12(2):184. doi: 10.3390/nano12020184 (PMC8780577; doi:10.3390/nano12020184)
Supplement: Supplementary file 1 [file nanomaterials-12-00184-s001.zip › nanomaterials-1520280-supplementary.pdf]

Supplementary information: Muon irradiation of ZnO rods: Superparamagnetic nature induced by defects

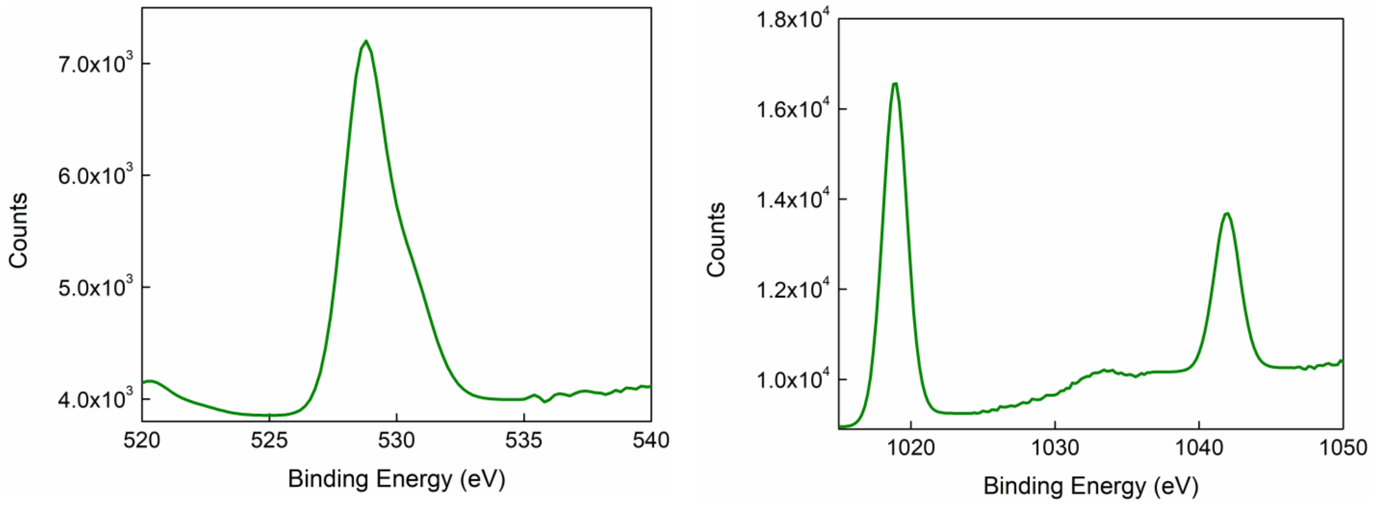

(a) (b)

Figure S1: XPS of sample 1 ZnO rods. (**a**) shows the binding energy (eV) for the different oxygen species. (**b**) shows the binding energy (eV) for the different Zn species.

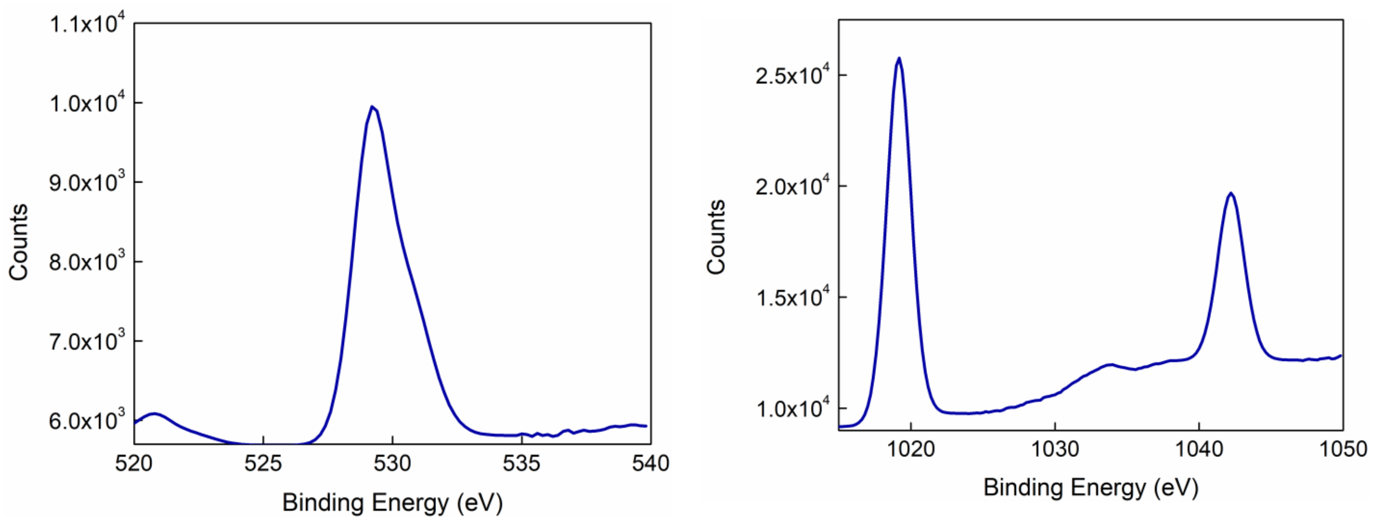

(a) (b)

Figure S2: XPS of sample 2 ZnO rods. (**a**) shows the binding energy (eV) for the different oxygen species. (**b**) shows the binding energy (eV) for the different Zn species.
